# Immunotherapy with an HIV-DNA Vaccine in Children and Adults 

**DOI:** 10.3390/vaccines2030563

**Published:** 2014-07-17

**Authors:** Paolo Palma, Lindvi Gudmundsdotter, Andrea Finocchi, Lars E. Eriksson, Nadia Mora, Veronica Santilli, Angela Aquilani, Emma C. Manno, Paola Zangari, Maria Luisa Romiti, Carla Montesano, Alba Grifoni, Andreas Brave, Karl Ljungberg, Pontus Blomberg, Stefania Bernardi, Eric Sandström, Bo Hejdeman, Paolo Rossi, Britta Wahren

**Affiliations:** 1 University Department of Pediatrics (DPUO), Unit of Immune and Infectious Diseases, Children’s Hospital Bambino Gesù, Piazza S. Onofrio 4, 00165 Rome, Italy; E-Mails: andrea.finocchi@opbg.net (A.F.); stefania.bernardi@opbg.net (S.B.); paolo.rossi@opbg.net (P.R.); 2 Chair of Pediatrics, Department of System Medicine, University of Rome Tor Vergata, 00133 Rome, Italy; E-Mails: nadia.mora@hotmail.it (M.N.); veronica.santilli@hotmail.it (V.S.); angela.aquilani@libero.it (A.A.); emma_m@hotmail.it (E.C.M.); paolazangari@hotmail.com (P.Z.); romiti@med.uniroma2.it (M.L.R.); 3 Department of Microbiology, Tumor and Cell Biology, Karolinska Institutet, Stockholm 171 77, Sweden; E-Mails: Lindvi.Gudmundsdotter@affibody.se (L.G.); andreas.brave@folkhalsomyndigheten.se (A.B.); karl.ljungberg@ki.se (K.L.); Britta.Wahren@ki.se (B.W.); 4 Affibody AB, 17163 Solna, Sweden; 5 Department of Neurobiology, Care Sciences and Society, Karolinska Institutet, Huddinge 171 77, Sweden; E-Mail: Lars.Eriksson@ki.se; 6 Department of Infectious Diseases, Karolinska University Hospital, Stockholm 171 77, Sweden; 7 School of Health Sciences, City University London, London EC1V 0HB, UK; 8 Department of Biology, University of Rome Tor Vergata, 00133 Rome, Italy; E-Mails: montesano@uniroma2.it (C.M.); alba.grifoni@uniroma2.it (A.G.); 9 Vecura, Clinical Research Center, Karolinska University Hospital, Stockholm 141 86, Sweden; E-Mail: pontus.blomberg@karolinska.se; 10 Venhälsan, Karolinska Institutet (KI), Södersjukhuset, Stockholm 118 83, Sweden; E-Mails: eric.sandstrom@sodersjukhuset.se (E.S.); bo.hejdeman@sodersjukhuset.se (B.H.)

**Keywords:** HIV-1, DNA vaccine, children and adults

## Abstract

Therapeutic HIV immunization is intended to induce new HIV-specific cellular immune responses and to reduce viral load, possibly permitting extended periods without antiretroviral drugs. A multigene, multi-subtype A, B, C HIV-DNA vaccine (HIVIS) has been used in clinical trials in both children and adults with the aim of improving and broadening the infected individuals’ immune responses. Despite the different country locations, different regimens and the necessary variations in assays performed, this is, to our knowledge, the first attempt to compare children’s and adults’ responses to a particular HIV vaccine. Ten vertically HIV-infected children aged 4–16 years were immunized during antiretroviral therapy (ART). Another ten children were blindly recruited as controls. Both groups continued their antiretroviral treatment during and after vaccinations. Twelve chronically HIV-infected adults were vaccinated, followed by repeated structured therapy interruptions (STI) of their antiretroviral treatment. The adult group included four controls, receiving placebo vaccinations. The HIV-DNA vaccine was generally well tolerated, and no serious adverse events were registered in any group. In the HIV-infected children, an increased specific immune response to Gag and RT proteins was detected by antigen-specific lymphoproliferation. Moreover, the frequency of HIV-specific CD8+ T-cell lymphocytes releasing perforin was significantly higher in the vaccinees than the controls. In the HIV-infected adults, increased CD8+ T-cell responses to Gag, RT and viral protease peptides were detected. No augmentation of HIV-specific lymphoproliferative responses were detected in adults after vaccination. In conclusion, the HIV-DNA vaccine can elicit new HIV-specific cellular immune responses, particularly to Gag antigens, in both HIV-infected children and adults. Vaccinated children mounted transient new HIV-specific immune responses, including both CD4+ T-cell lymphoproliferation and late CD8+ T-cell responses. In the adult cohort, primarily CD8+ T-cell responses related to MHC class I alleles were noted. However, no clinical benefits with respect to viral load reduction were ascribable to the vaccinations alone. No severe adverse effects related to the vaccine were found in either cohort, and no virological failures or drug resistances were detected.

## 1. Introduction

Highly active antiretroviral therapy (ART) is the principal tool to control HIV infection [1]. The benefits of ART regimens in children and adults are widely demonstrated in terms of viral control and immune reconstitution [2]. The main persisting problems are the life-long need for strict therapeutic adherence, the emergence of side effects, the risk of developing resistance and the cost of medication [3,4,5,6].

In the clinical context, new therapeutic approaches, such as HIV vaccines, have been investigated in recent years in both children and adults [1,7,8,9,10,11]. The goal of immune intervention in HIV infection is to restore immunocompetence and reverse the anergy-like situation in infected individuals. This, in turn, could reduce viral load and the virus-producing cells in viral reservoirs, permitting extended periods without ART. Therapeutic vaccines have been tested extensively, such as envelope proteins [12,13,14], killed virus preparations [15,16], pox immunogens, such as ALVAC (a replication-deficient vaccine vector derived from canary pox) [17], peptides [18] and HIV-expressing DNA plasmids [9,19,20,21,22,23,24,25]. The only one of these trials to indicate a clinical effect and short-term improved survival was repeated gp160 glycoprotein immunization, including 835 HIV-infected individuals on no or low-efficiency antiretrovirals [14]. 

DNA vaccines mimic live attenuated vaccines, inducing innate, humoral and cellular immune responses, without the risks associated with the administration of infectious or attenuated agents. The multigene, multi-subtype HIV-DNA vaccine (HIVIS) is a combination of plasmid DNA constructs, encoding several components, including structural and non-structural genes from several subtypes of HIV-1 [26]. This HIV-DNA has been studied extensively in terms of preclinical safety and immunogenicity [27,28,29,30,31].

The objective of the present study is to compare the findings obtained in two principal trials conducted in HIV vertically-infected children and chronically-infected adults in terms of feasibility, safety and immunogenicity. Despite the different country locations, different regimens and the necessary variations in assays performed, this is, to our knowledge, the first attempt to compare children’s and adults’ responses to a particular HIV vaccine.

## 2. Experimental

### 2.1. Study Subjects and Trial Design

The PEDVAC (acronym for our paediatric HIV vaccine trial) trial included twenty HIV-1 subtype B vertically-infected children (4–16 years of age) on stable antiretroviral regimen for at least 6 months with HIV-1 RNA <50 copies/mL and stable CD4+ T-cell counts (≥400 cells/mm^3^ or 25%) over 12 months of follow-up (Table 1). Patients were randomized into two arms: a control group of 10 children who continued their previous antiretroviral regimen (Arm A) and received no placebo, as determined by the local ethical committee, and a group of 10 children immunized intramuscularly with the HIV-DNA vaccine in addition to their previous ART regimen (Arm B). In the PEDVAC trial, the patients were kept on ART for the duration of the study [9]. In the children, immunizations were administered by intramuscular injection at Weeks 0, 4 and 12 with a boosting dose at Week 36.

In the adult study (Dermavax), twelve chronically HIV-1 subtype B-infected male patients (29–56 years of age) on an antiretroviral regimen (ART) with viral loads of <50 copies/mL and CD4+ T-cell counts of >450 cells/mm^3^ for at least 6 months were enrolled (Table 1). The adults were randomized into three groups, modelled on a successful study in macaques [32]: a control group of 4 patients (Group 1), a group of 5 patients immunized with HIV-DNA plasmids (Group 2) and a group of 3 subjects immunized with HIV-DNA plasmids and treated orally with hydroxyurea daily (Group 3) until Visit 10 [22]. Hydroxyurea was added to decrease immune activation, but in order to identify vaccine-specific reactivities, Groups 2 and 3 were handled as a single group of 8 vaccinated adults. The immunizations were performed during three cycles of 7 weeks of ART followed by four weeks of therapy interruption. In adults, dermal vaccinations were given at Weeks 4, 6, 15, 17, 26 and 28. After the third cycle of ART, including the last two immunizations, the patients were allowed to continue long-term treatment interruption until they showed signs of immunological or virological failure. In both cohorts, HIV viral load, CD4+ T-cell count and other immunological samplings were periodically performed.

**Table 1:** Demographic characteristics of paediatric and adult cohorts.

Characteristics	Paediatric Study (*N* = 20)	Adult Study (*N* = 12)
Age ± SD, year	11.6 ± 2.6	41.7 ± 6.8
Sex, no. male/female	7/13	12/0
Caucasian race, no. (%)	18 (90%)	10 (83%)
Placebo/HIV-DNA vaccine, no.	10/10	4/8
CD4+ T-cells/mm^3^ at baseline, mean ± SD	763.6 ± 231.1	685.9 ± 137.7
CD4+ T-cells/mm^3^ at last visit, mean ± SD	803.1 ± 237.5	545.4 ± 148.1
HIV-RNA level at baseline copies/mL, mean	<50	<50
HIV-RNA level at last visit copies/mL, mean	<50	<50
Duration of vaccination: duration of study weeks	36:96	28:60
Antiretroviral therapy during study, weeks	96	21
Observation period, weeks	156	300

### 2.2. Vaccine Formulation and Administration Technology

In the PEDVAC trial, two preparations of HIV-DNA plasmids were used: (1) DNA plasmids (2 mg/mL) encoding HIV Env A, B, C and Rev B in Ampoule 1; and (2) DNA plasmids (2 mg/mL) encoding HIV gp 160 Gag A, B and RTmut B in Ampoule 2. The development of the expression vectors containing the HIV-1 envelope genes *gp160 A*, *B* and *C* (pKCMVgp160A, pKCMVgp160B, pKCMVgp160C); *rev* (pKCMVrev); reverse transcriptase *RTmut* (pKCMVRTmut); *p37BA* and *p37B* (pKCMVp37BA, pKCMVp37B) were previously described [29,33,34].

Each child received 1.2 mL of *Env-Rev DNA* (from Ampoule 1) in the left arm and 0.8 mL of *Gag-RTmut DNA* (from Ampoule 2) in the right arm, amounting to a total of 4 mg of HIV-DNA by intramuscular injection at the two deltoid sites at each visit. In total, each child received 16 mg HIV-DNA given by needle intramuscularly.

The HIV-DNA vaccine plasmids used in the adult study were the same combinations of: (1) gp160 subtypes A, B and C together with Rev subtype B; and (2) Gag subtypes A and B together with RTmut subtype B (see above). The plasmid combinations were delivered separately, formulated in polyethylene imine mannose (PEIm) and dextrose solution [35,36]. For each vaccination, 3.2 mL vaccine (a total amount 0.4 mg at each visit) or placebo (only formulation solution) were administered at four skin sites that had been freed of the superficial layer of keratinocytes [22,37]. In total, each adult received 2.4 mg of HIV-DNA given directly on large areas of scraped skin, intended to directly reach dendritic cells [37].

### 2.3. Safety Evaluation

In the paediatric study, patients or their parents recorded, in a self-administered diary card, the occurrence and severity of solicited local and systemic events, using a standard scale to grade reactions. In both studies, physical safety evaluations were performed before vaccination and at each visit. Immediate local and systemic reactions were observed during the first hour after the HIV-DNA application. All medical events were recorded as possible adverse events and were graded by the principal investigator as to their severity and relationship to the immunization. The standard AIDS Clinical Trials Group (ACTG) grading scale was used to evaluate adverse events in both studies.

### 2.4. Laboratory Assays

#### 2.4.1. CD4+ T-Cell Counts, Plasma HIV-1 RNA Levels and Cell-Associated HIV-DNA Quantification

The CD4+ T-cell number in blood was measured by flow cytometry. Viral load in plasma was measured by the Quantiplex HIVRNA 2.0 bDNA Assay, Chiron Diagnostic Corporation, Emeryville, CA, USA, and the viral load of lymphocytes by HIV-DNA quantification. In addition, an ultrasensitive method was used to quantify virus down to 1 viral RNA copy/mL. The plasma was pelleted, the pellet virus extracted in 1/8 of the original volume and assayed by reverse transcription and PCR following the method for Amplicor HIV-1 monitor v. 1.5 (Roche Molecular Systems, Pleasanton, CA, USA). In plasma of adults, the Roche Diagnostic System with a lower limit quantification of 50 copies/mL of plasma viral load was used.

#### 2.4.2. Cellular Immune Responses

In both studies, the immune response to HIV-DNA vaccine was evaluated by lymphoproliferation and other functional assays. Lymphoproliferation assays were performed in both adults and children on fresh peripheral blood mononuclear cells (PBMC) cultured with HIV antigens and mitogens, as described below [9,22].

Briefly, in children, PBMC were cultured in 96-well plates for 7 days with aldrithiol-2 (AT-2)-treated HIV-1 MN virions subtype B at 2.5 µg/mL and non-viral control antigen SUPT1 microvesicles at 2.5 µg/mL (kindly provided by Dr. J. Lifson, SAIC Frederick, Inc., Frederick, MD, USA), recombinant reverse transcriptase (rRT) subtype B at 0.5 µg/mL, rp24 at 0.5 µg/mL and recall antigen cytomegalovirus (CMV) at 0.5 µg/mL and *Candida albicans* at 0.5 µg/mL (Nanogen, Torino, Italy). Results are shown as mean values of duplicates and are expressed as counts per minute (cpm). The stimulation index (SI) was defined as previously described [9,38] and was considered to be positive when higher than 3.

In the paediatric cohort, functional tests further consisted of intracellular staining (ICS) after stimulation with a combination of HIV-1 proteins [9]. Heparinized blood was incubated with 1 µg each of anti-CD28 and anti-CD49d monoclonal antibodies (mabs) and 1 µg of a protein pool consisting of recombinant proteins representing the vaccine sequences of HIV-1 RT (Clade B), HIV-1 p17/24 (Clade B), HIV-1 p17/24 (Clade C) or mabs only, for 16 h. After 2 h, 10 μg/mL of brefeldin A (Sigma-Aldrich, St. Louis, MO, USA) were added. After 14 h, cells were stained with anti-CD3, anti-CD4 and anti-CD8 antibodies. Red blood cells were lysed with BD FACS Lysing solution (BD, San José, CA, USA), fixed with 4% paraformaldehyde and washed. Lymphocytes were stained with anti-IFN-γ mab and analysed with a FacsCanto II. Results were expressed as the percentage of cytokine-producing CD3+, CD4+ and CD8+ T-cells. A percentage of cytokine-secreting cells of HIV-stimulated cultures higher than or equal to 0.02% above the unstimulated cultures was considered positive. A percentage of cytokine-producing control cells lower than 0.06% was requested for evaluation [9,38].

In the adult cohort, ELISpot was performed with HIV Env, Gag and Pol peptide pools (NIH repository, MD) [22]. ELISpot assays were performed with pre-coated anti-IFN-γ and anti-perforin ELISpot plates (Mabtech, Nacka Strand, Sweden), using fresh PBMC and PBMC depleted of CD8+ T-cells. HIV-1 subtype B peptide pools corresponding to Env, Gag and Pol (NIH repository, MD) were used at 2.5 µg/mL. Plates were incubated for 22 h for IFN-γ ELISpot and 44 h for perforin ELISpot at 37 °C). The results were expressed as the mean number of spot-forming cells (SFC) per 10^6^ PBMCs. ELISpot responses were considered positive if the number of spot-forming cells was >4 times the background and >55 SFC/10^6^ PBMCs.

#### 2.4.3. Humoral Immune Responses

Binding antigen-specific antibodies to HIV-1 subtype B gp160 (Protein Sciences Corporation, Meriden, CT, USA) or recombinant HIV-1 subtype B p24 Gag (CFAR, Potters Bar, UK) were evaluated in ELISA-based assays for both cohorts [11].

### 2.5. Ethics

The protocols were approved by the Ethical Committee of the Children’s Hospital Bambino Gesù of Rome and by the Karolinska Institutet of Stockholm, for the paediatric and adult studies, respectively. Written informed consent was obtained from all participants and/or their parents.

### 2.6. Statistical Analysis

Statistical calculations were performed in GraphPad Prism Software version 5.00 and IBM SPSS Statistics v. 22. Statistical significance was accepted if *p* < 0.05. Comparisons between the paediatric and adult cohorts were calculated by Mann–Whitney *U* tests within cohorts and between cohorts.

## 3. Results

### 3.1. Patients Enrolled

The characteristics of the two cohorts are presented in Table 1.

### 3.2. Safety

The HIV-DNA vaccine was generally well tolerated, and only one serious adverse event was detected during the studies in the adult cohort (Table 2).

**Table 2:** Reported adverse events of vaccinees.

	Grade 1		Grade 2		Grade 3	
	Local	Systemic	Local	Systemic	Local	Systemic
Vaccinated children (*n* = 10)	12	16	2	6	0	0
Vaccinated adults (*n* = 8)	39	17	0	4	1	0

**Figure A1:**
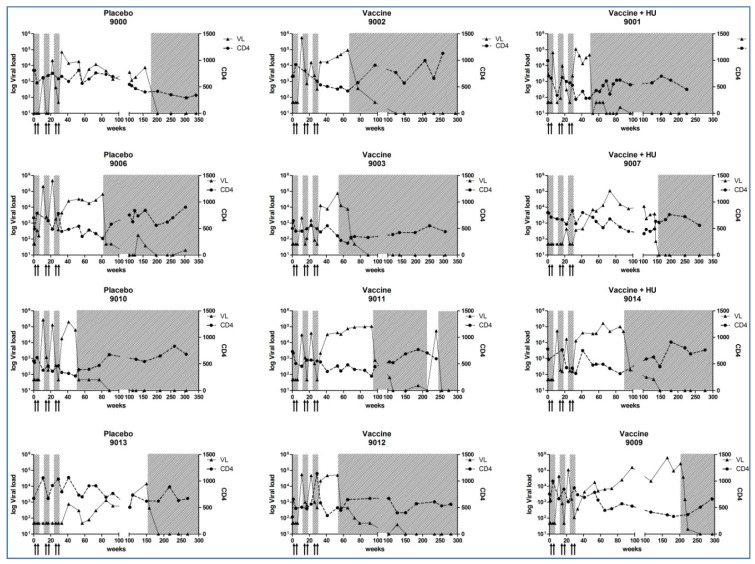
Schedule of 12 adult vaccinees. CD4+ T-cells (bullets) and HIV-1 viral load (triangles) during vaccination (arrows) and repeated structured therapy interruption (STI) (white fields). All patients finally resumed antiretroviral therapy (shaded areas).

In the paediatric study, a total of 36 adverse events related to vaccination were recorded from the first injection to one month after the last one with an excess of local events in the vaccine group. All reactions in children or adults were graded as mild or moderate, and no accumulation of adverse events was registered. In the vaccinated adult group, a total of 61 adverse events were detected during the study. Only one Grade 3 event was registered in a patient in the adult cohort (Table 2). This was a rash attributed to hydroxyurea treatment. This patient was continued on vaccine only [22]. The duration until ART was re-initiated after the third vaccination and STI cycle, was 15 months for vaccinated (range 20–185 weeks) and 18 months for placebo (range 20–145 weeks), a non-significant difference (Appendix Figure A1). None of the paediatric or adult participants failed any vaccination.

### 3.3. CD4+ T-Cell Counts, Plasma HIV-1 RNA Levels and Cell-Associated HIV-DNA Quantification

In the paediatric cohort, no patient experienced virological failure. In three of the twenty children included (one in the control group and two in the vaccinated group), a single viral blip was detected (not exceeding 1000 copies/mL of plasma HIV-RNA), which returned to below 50 copies/mL of viral RNA at the following determination. CD4+ T-cell counts remained above 400 cells/mm^3^ over time.

In the adult cohort, an increased plasma viral load was observed during the STI phases and after the final interruption of ART in all, except two patients (one placebo and one vaccinee), the viral loads of whom remained low during the short-term STIs (Appendix Figure A1). The viral peaks during STIs ranged from below 50 viral genomes/mL to 105 viral genomes/mL, and virus replication recurred during the second and third STIs (Appendix Figure A1). The differences between the highest levels (peaks) of virus during the three cycles of STI were not statistically significant between the vaccine and the placebo group. Of particular interest is that the plateau levels of virus after the final third STI at months 1, 2, 3, 4, 5 were significantly lower than the viral set points before the first initiation of antiretroviral therapy for the whole adult group (p = 0.003) [22]. The mean viral set point for all adults was 105, while median levels following the last STI slowly increased from 104.1 to 104.4 viral genomes/mL. The viral levels following the last STI, thus, had significantly decreased, when compared to the original viral set point. Viral levels were calculated as median values of consecutive measurements following the third STI ([22] and Appendix Figure A1).

We have previously shown that drug-resistance did not develop [22]. As expected, the CD4+ T-cell counts decreased during the first two STIs and these levels were comparable in the vaccinated and placebo groups. All patients finally resumed antiretroviral therapy without significant time differences between placebo and vaccinees (Appendix Figure A1).

### 3.4. HIV-Specific Immune Responses

#### 3.4.1. Cellular Immune Responses

Smaller volumes of samples could be obtained from children compared to adults, and the frequency of sampling was lower in the child cohort. It is also likely that the results of a particular assay may vary between sites. We have therefore compared immune responses to the baseline responses of each cohort, rather than comparing actual values between cohorts.

**Figure 1:**
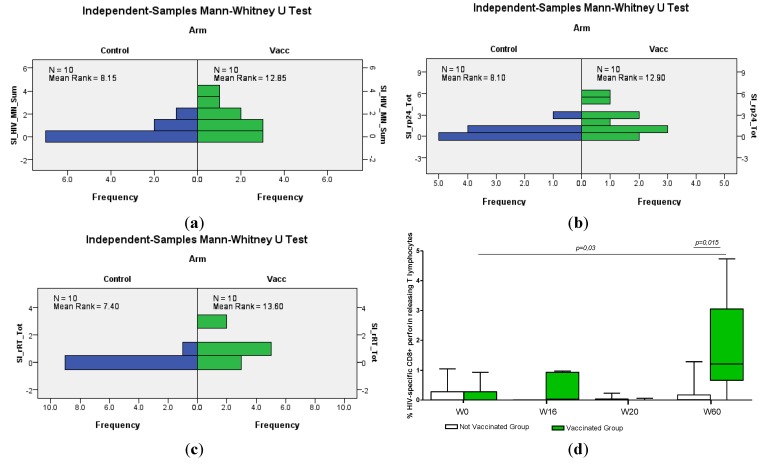
Lymphocyte proliferation assays of the paediatric cohort, showing responses to (**a**) the HIV-1 MN antigen; (**b**) the p24 antigen; (**c**) the RT antigen (summarized, sum or total, for Weeks 12–72); (**d**) the intracellular staining (ICS) data of perforin-releasing CD8+ T-cells are shown.

In the paediatric cohort, higher lymphoproliferative responses to Gag proteins (HIV-1 MN and p24, both representing HIV-1 Gag subtype B) were observed in the vaccinees compared to controls (Figure 1a, b).

By identifying individuals with a high stimulation index (SI) over 15, we found that 7/10 vaccinated and 3/10 non-vaccinated children present a positive lymphoproliferative response detectable at one or more occasions to the HIV-1 MN antigen after the immunization period from Week 12 to Week 72 (*p* = 0.055, Figure 1). At 16 weeks, vaccinees, but no controls, had a high SI to HIV p24 (*p* = 0.073). SI values to RT after vaccination were positive at least once in 7/10 vaccinated and in 1/10 non-vaccinated children (*p* = 0.007, Mann–Whitney *U* test). Such lymphoproliferative responses were transient, and the highest SI for these infected children was 30. In healthy vaccinated individuals, a maximum SI of around 30 was noted after HIV-DNA immunization [39].

**Figure 2:**
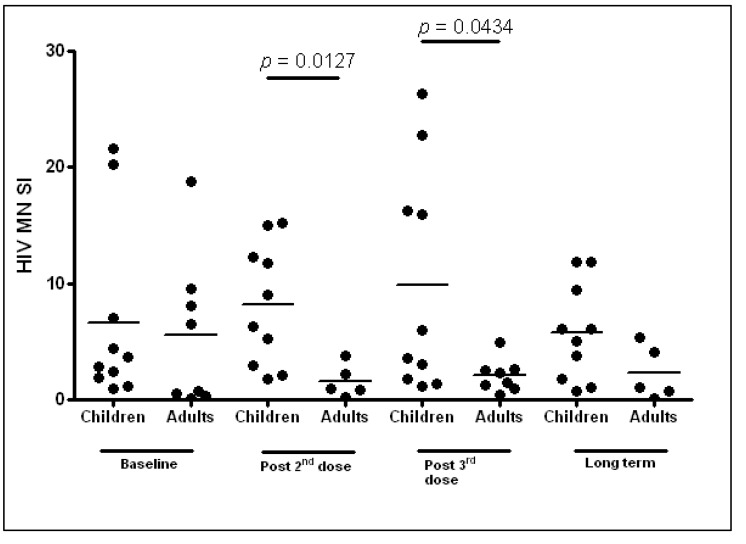
Comparative HIV-1 MN antigen lymphoproliferation for paediatric and adult cohorts. SI, stimulation index.

In the adult cohort, no changes in lymphoproliferative responses to HIV-1 antigen MN (representing mainly Gag subtype B) were detected after any of the vaccine doses or during the therapy interruption period. Comparisons between the paediatric and adult HIV-1 MN lymphoproliferative responses are shown in Figure 2.

To estimate CD8+ T-cell responses, ELISpot IFN-γ and intracellular staining (ICS) assays were performed. The frequency of HIV-specific CD8+ T-cell lymphocytes releasing perforin after stimulation with a combination of Gag and RT proteins measured by ICS was significantly higher in the vaccinated paediatric group than in non-vaccinated children (Figure 1d). This occurred after the third HIV-DNA vaccine dose, compared to baseline. In the vaccinated paediatric group, a significant increase of CD8+ T-cell perforin-releasing cells was also observed after the last boosting immunization compared to baseline (Figure 1d). Thus, CD8+ T-cell perforin levels as measured by ICS were elevated late in vaccinated individuals; four of the vaccinated children had repeatedly elevated perforin-secreting cells after vaccination. Low or no ELISpot reactivity was noted by measuring IFN-γ in response to single Gag, Env or RT peptides.

**Figure 3:**
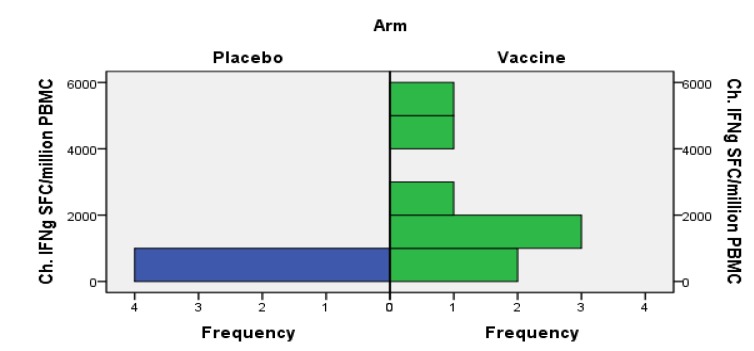
Increases of HIV-specific and recall-induced IFN-γ ELISpot responses in placebo and vaccine recipients in the adult cohort. Changes (Ch.) in HIV-specific IFN-γ (IFNg) responses to Gag, RT and protease peptides at visit 10 are shown. The baseline responses have been subtracted. The vaccine group had more prominent increases of HIV-specific IFN-γ responses of peptide reactivities than the placebo group (Mann–Whitney *U* test, *p* = 0.028).

The adult vaccine group had a higher net gain of HIV-specific IFN-γ responses from baseline to Week 10 compared to the placebo group (*p* = 0.028, Figure 3). The levels of perforin-secreting cells were high at baseline. There were distinct increases of peptide-specific responses as measured by ELISpot IFN-γ to peptides representing HIV-1 subtype B. Altogether, 28 responses occurred in the vaccinated cohort and 11 in the placebo group. They were tentatively allocated to alleles HLA (Human Leucocyte Antigen) A*0201, A*0301, B*35 and B*5701, among others, and occurred for several peptides summarized to represent Gag, RT and viral protease PR (Figure 3) [22]. We must therefore conclude that vaccination, together with STI, permitting viral stimulation, contributed to the detection of the novel HIV-specific CD8+ T-cell responses. 

#### 3.4.2. Humoral Responses

In children, the vaccination protocol did not appear to affect binding antibody levels. In the adult cohort, gp160 and p24 titres were high and remained at the same level in all, except two vaccinated patients, who significantly increased their titres to both Env and Gag protein antigens. These two individuals raised their antibody titre to gp160 subtype B 20- or 100-fold and antibody responses to Gag p24 subtype B 10- or 300-fold, respectively.

## 4. Discussion

This is a comparative study focusing on HIV-DNA vaccination in infected children and adults. Both the paediatric and the adult trials separately demonstrated feasibility, safety and moderate immunogenicity [9,11,22]. Very importantly, genetic vaccination was not observed to increase virus amounts, as had been feared [40]. We can thus conclude that the HIV-DNA vaccine, called HIVIS, is a safe vaccine strategy in paediatric and adult HIV-infected individuals. We have not observed increased viral replication above what was reported in non-vaccinated controls. No major adverse events occurred during or after the vaccination schedules, either in paediatric or in adult cohorts, confirming the safety profile of genetic vaccination, also reported in healthy subjects [39,41].

In children, due mainly to a more preserved immune system and to the continuation of ART during the study period, a good virological control and maintenance of CD4+ T-cell values were observed compared to the adult study. Our sensitive viral RNA and DNA assays, however, could measure very low viral loads, varying around 1–10 RNA viral copies/mL of blood of both vaccinated and control children. Thus, the HIV-specific immune response did not significantly influence viral replication rates during ART, even at very low viral levels.

In contrast, in the adult cohort, a rebound of plasma viral load and decreased CD4+ T-cell levels during STI periods were observed, which were similar to that of the adult placebo patients. Following the last STI, all adults had quite a long period without drug, a median of 15–18 months, similar for vaccinated and placebo recipients. The cycles of treatment interruptions in the adult cohort induced changes in several parameters of innate immunity. We noted increased TLR7/8-triggered secretion of IL-12 and IFN-α, as well as TLR9-triggered secretion of IL-12. On the other hand, we noted a reduction of TLR9 stimulated IFN-α plasmocytic and myeloid dendritic cells [42]. These results suggest that the consequences of short-term STI include dysregulated TLR responses and fluctuations in the frequencies of circulating dendritic cells. Possibly these factors, related to innate immunity, contributed to the rapid increase in viral replication in all, except two, adult individuals during the first two STIs.

In HIV-infected children and adults, novel antigen-specific cellular immune responses were elicited after vaccination. In the HIV-infected children, we noted increased specific cellular immune responses to Gag antigens (HIV-1 strain MN and p24) and to RT antigens. These reactions were detected by lymphoproliferation assays after vaccination, indicating primarily a CD4+ T-cell-specific response. Gag-specific T-cell responses have been correlated with lower levels of viral load in chronically infected patients [43,44,45,46]. Lymphoproliferative responses were, however, not observed in the adult cohort. The difference in antigen-specific lymphoproliferation responses between the children and adults may be due to the length of exposure to the endogenous viral infection, which, untreated, had occurred for a longer time in the adults.

In some studies, RT-specific T-cell responses have been reported to be of protective value in HIV-infected individuals [47,48]. In children, RT lymphoproliferative responses were detected after vaccination, whereas no such reactivities were induced by the same HIV-DNA vaccine in healthy adults. The lymphocytes of vaccinated children appeared more reactive with RT proteins (CD4+ T-cell related) than vaccinated adults, who instead responded better to RT peptides (CD8+ T-cell related). This phenomenon might be due to a more intact immunological status of the children in this study [49]. According to worldwide HIV treatment guidelines, the children’s cohort started ART during the first year of life, while the adults started ART when CD4+ T-cell values were below 350 cells/mm^3^. The delayed antiretroviral therapy in adults thus results in a more pronounced immune exhaustion compared to early-treated children.

In the paediatric cohort, an increase of HIV-specific CD8+ T-cells secreting perforin was detected after three vaccine doses and after the late boosting with HIV-DNA. Novel CD8+ T-cell-specific responses to HIV Gag and RT were seen in infected vaccinated adults and could be typed to identify responding alleles [22].

No significant increases of antigen-specific humoral responses were detected in children and in only two of the adults after vaccinations. These data are disappointing, but consistent with the performance of other stand-alone DNA vaccines delivered to date. In healthy adults, on the other hand, priming with this HIV-DNA vaccine used in combination with vaccinia-derived boosts, has evoked potent humoral responses after two HIV-MVA boosts [39,41,50]. 

The functional immune responses thus appear to be different in the two cohorts, probably reflecting many parameters, such as age, the duration of the HIV infection, the timing of ART initiation and the duration of the ART. With increasing age, the human immune system undergoes characteristic changes, termed immunosenescence, leading to insufficient protection following vaccination [51]. Immunosenescence is also observed in chronic HIV infection [52,53,54]. Therefore, biological age and the duration of HIV infection negatively influence the immune responses in the adult cohort, in addition to long periods with high viral loads before therapy, as mentioned above [55].

There are possibilities to increase the immunological pressure against virus-producing cells. One might apply a prime-boost immunization schedule with HIV-DNA followed by modified vaccinia-based boosts (HIV-MVA), which has been highly successful to induce both cellular and humoral immune responses in healthy adults [39,41,50]. In neither our childhood cohort nor in the adult cohort was it considered safe or ethical at that time to progress with attenuated vaccinia-based immunotherapy. Another boosting strategy may be a protein, which would primarily induce CD4+ T-cell and B-cell responses.

The aims of the present studies were to identify modes by which repeated immunizations can work together with, or substitute for, drugs during certain sensitive periods in the life of the HIV-infected individual. Young adults, particularly in adolescence, could benefit from periods of reduced treatment [56]. Such attempts must be monitored closely. 

## 5. Conclusions

In conclusion, the HIV-DNA vaccine can elicit new HIV-specific cellular immune responses, particularly to Gag antigens, in infected children and adults, albeit with different profiles. Vaccinated HIV-infected children mounted a CD4+ T-cell-related HIV-specific immune response, whereas the infected adults primarily showed CD8+ T-cell responses related to their MHC class I alleles. None of these immunization schedules was potent enough to change the viral load dynamics over time or during therapy interruptions in HIV-infected adults. Additional studies and new virological tools are required to evaluate the impact of vaccination on the viral reservoirs in children before periods of ART interruption are attempted in this population.
